# Crystal Growth Kinetics of GeSe_2_ Polymorphs in Bulk Glasses and Thin Films: Role of Self-Diffusion and Viscosity

**DOI:** 10.1021/acs.cgd.5c01063

**Published:** 2025-09-13

**Authors:** David Vaculík, Jaroslav Barták, Simona Martinková, Petr Koštál, Jiri Málek

**Affiliations:** † Department of Physical Chemistry, 48252 University of Pardubice, Studentská 573, Pardubice 532 10, Czech Republic; ‡ Department of Inorganic Technology, University of Pardubice, Doubravice 41, Pardubice 532 10, Czech Republic

## Abstract

The knowledge of transport properties (viscosity and self-diffusion) and the knowledge of crystal growth of different polymorphs in amorphous materials prepared in different forms provide important information for the preparation, processing, and utilization of these materials. This article is the first study of the direct observation of crystal growth rates in amorphous GeSe_2_ bulk samples and thin films. The study contains a detailed analysis of viscosity and crystal growth in amorphous GeSe_2_ samples (bulks and thin films), revealing also information about the self-diffusion process. Two polymorphs of GeSe_2_ crystals (low temperature—LT, and high temperature—HT) grew in GeSe_2_ bulk glasses. In the thermal evaporated film, only LT-GeSe_2_ was found. Nevertheless, the crystals in thin films grew far below the glass transition temperature. To properly analyze and describe the crystal growth kinetics, viscosity data were obtained using a thermomechanical analyzer and a nanoindentation system. A combination of crystal growth data and viscosities provides information about the size and transport speed (self-diffusion) of structural units incorporated into the GeSe_2_ crystals.

## Graphical abstract

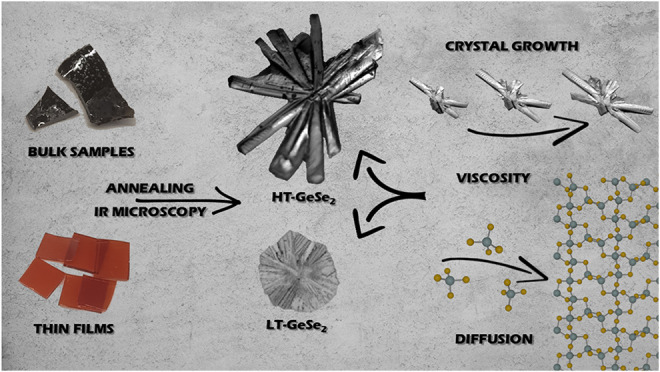

## Introduction

Chalcogenide materials are known for their optical and electrical properties,^1^ which makes them useful for photonic and electronic applications. Germanium diselenide is a two-dimensional (2D) material with high in-plane anisotropy and a wide band gap of 2.74 eV, which recently attracted much attention in the research of polarization-sensitive photodetection in short-wave regions^2,3^ and also for their high energy density as an anode material for lithium-ion batteries.^4,5^

The literature reports up to eight structural forms of GeSe_2_, with three stable crystalline phases.^6–15^ The orthorhombic α-GeSe_2_ with the *Pmmm* or *Pmn* orientation space group is also known as the low-temperature modification (LT-GeSe_2_) and has a complex structure growing in three dimensions^6,16,17^ with the character of a distorted CdI_2_-type lattice.^6^ The most stable phase, the monoclinic β-GeSe_2_ modification, is denoted as the high-temperature modification (HT-GeSe_2_). This in-plane structure has the space group of *P*2_1_/*c* and is isotypic to the α-GeS_2_.^18^ The corners connect the tetrahedra into a [GeSe_4_]*_n_* chain along the *a* axis, which is also connected by the edges of [Ge_2_Se_8_] double tetrahedral units along the *b* axis, creating planar crystal structures.^9,19^ The unit-cell parameters of β-GeSe_2_ are *a* = 7.016 Å, *b* = 16.796 Å, *c* = 11.831 Å, and β = 90.65°, and the volume of the cell is 1394 Å^3^.^15^ The main difference between the α and β modifications is that the β modification has equal amounts of corner and edge-sharing tetrahedra, but the α modification consists only of corner-sharing units.^2^ The third stable modification is hexagonal γ-GeSe_2_, with its structure being related to the SnSe_2_-type lattice. This modification differs from the previous two because it exhibits in-plane isotropy.^2,6^

Various studies have thoroughly investigated the structure of germanium diselenide glass^20,21^ using X-ray or neutron diffraction methods. These studies imply that GeSe_2_ glass and liquid phases are composed of GeSe_4_ tetrahedral units. The short-range ordering in the amorphous phase shows the presence of edge and corner-shared GeSe_4_ tetrahedra,^20^ similar to the crystalline phase. The tetrahedral units also have a considerably broken-up structure with similar but not exact cell parameters as in the crystalline structure. The bond angle between neighboring corner-shared tetrahedra slightly differs, as do the lengths of the bonds between Ge–Ge and Ge–Se. Only the Se–Se bonds are very similar in crystalline, glass, and liquid phases.^21^ Vashishta et al.^22^ presented results with the use of molecular-dynamics calculations that show the angle between the Se–Ge–Se distribution to be 106°, the Ge–Se–Se distribution around 37°, and an internal angle at the Se–Se–Se distribution to be 57°, which further proves the presence of distorted tetrahedra structures in liquid and glassy phases of germanium diselenide.

This work aims to provide a detailed study of the crystal growth kinetics in stoichiometric GeSe_2_ bulk glass and thin films above and below the glass transition temperature. The difference in crystal growth character in bulk and thin film samples is discussed, as well as the viscosity behavior of both sample types. The crystal growth rate is combined with viscosity data and described by a suitable kinetic growth model. Analysis of the crystal growth model also brings insight into the diffusion process driving the crystal growth in the studied chalcogenide glass former.

## Materials and Methods

The bulk amorphous samples of GeSe_2_ were prepared from high-purity elements (5N, HiChem, Prague, CZ). These elements were weighed to 10 g of stoichiometric composition, placed into a silica glass ampule, and thoroughly cleaned with aqua regia. The ampule was then evacuated to a pressure of 10^–3^ Pa and sealed. After that, the evacuated and sealed ampule was put into the rocking furnace by VEZAS s.r.o. (Hradec Kralove, CZ) and heated at 1223 K to provide a good reaction between Ge and Se elements. The ampule was held at this temperature for 24 h, after which the temperature was lowered to 1073 K to reduce the vapor pressure and for safety reasons. After another 4 h, the ampule was quickly quenched in ice–water to ensure the amorphous character of the prepared chalcogenide glass. After opening the ampule, the as-prepared GeSe_2_ glass ingots of black color with a shining reflective surface were obtained. Part of these samples were used for kinetic experiments of crystal growth measurements, and the rest were used to prepare thin film samples. These samples were prepared using thermal deposition in the evacuated aperture at a pressure of 2·10^–4^ Pa with a deposition rate of 1–2 nm/s until the desired thin film thickness of 1000 nm was achieved. The deposition rate and thickness of the deposited layer were examined using the silica crystal microweighting method (MSV-1843/A MIKI-FFV). The microscope glass slides were used as a thin film substrate and thoroughly cleaned with isopropanol.

The composition of the prepared glass samples was verified with the use of an energy-dispersive X-ray microanalyzer (EDS Xflash 660H, 60 mm^2^, Quantax 75 EDS, Bruker Co.) coupled with a scanning electron microscope (SEM Hitachi TM 4000 II). The EDS analysis showed the composition of the as-prepared bulks to contain 32.41 ± 0.16 atom % Ge and 67.51 ± 0.16 atom % of Se. As-prepared thin films contained 31.61 ± 0.08 atom % of Ge and 68.39 ± 0.08 atom % of Se. The EDS analysis was performed on uncoated samples. The standard deviations show compositional variation in several tested places in several samples. Nevertheless, the accuracy in composition determination using the noted EDS detector is about 1 atom %.

An X-ray diffraction (XRD) analyzer (MiniFlex 600 HR with Bragg–Brentano θ–2θ geometry; Cu Kα λ = 1.54 Å, *U* = 40 kV, and *I* = 15 mA) was used to check the amorphous character of the as-prepared samples and to characterize the crystalline phase growing in the samples. The XRD scans were collected with the use of the ultrafast detector D/Tex Ultra 2 in the range of 2θ = 10–60°, scanning speed of 1–5°/min, and measuring step of 0.01°.

Differential scanning calorimetry (DSC, PerkinElmer Pyris 1 with an intercooler 2P) was used to measure the thermal behavior of the amorphous samples. Samples of scratched thin films (3.37 mg) and bulk (10.79 mg) were measured in standard aluminum pans under an atmosphere of dry nitrogen. The experiments were performed in the temperature range 473–653 K under a heating rate of 10 K/min. Bulk samples (approximately 60 mg) were also measured in open silica glass ampules at a 10 K/min heating rate using a DSC Sensys Evo three-dimensional (3D), Setaram Co. The use of silica ampules allows measurements up to 1073 K.

The kinetics of growing crystals were studied using the Olympus BX51 infrared microscope with a mounted XM10 digital camera. Since the samples were transparent in the infrared light spectrum, observing the crystals in both transmission and reflection modes was possible. The significant difference between the crystalline phases and the amorphous glass’s optical properties allowed a good evaluation of crystal growth behavior. Thin film samples were first heat-treated in a computer-controlled furnace and then evaluated under a microscope to study crystal growth at lower temperatures. TF samples were also assessed in real-time using a programmable annealing stage, Linkam, which is suitable for fast measurements at higher temperatures up to 873 K. Measurements of bulk samples, prepared by cutting synthesized glass ingots into smaller pieces, were performed only using the ex situ method in a computer-controlled furnace. After annealing, the samples were cleaved in half to observe the crystals growing in volume, although the surface crystallization was also studied. In both cases, the samples were annealed isothermally for diverse time intervals, with the whole thermal history recorded to check the isothermal character of the experiments.

The viscosity properties of chalcogenide glass samples were studied by using various methods. The thermomechanical analyzer (TMA CX03, RMI, Czech Republic, equipped with a high-precision differential capacitance displacement probe detector) was used to study viscosities in bulk samples. The aperture was calibrated on melting points of pure metal standards (Ga, In, Sn, Pb, Zn, and Al). The measurements were carried out with bulk samples of GeSe_2_ in the form of plates, which were ground on both sides to ensure the surfaces were parallel. Nanoindentation (NI) was another method used for measuring viscosity, which was performed using the Hysitron TI Premier nanoindenter, equipped with xSOL 600 heating stage, Bruker Nano Inc., with temperature stability ±0.2 K. For these measurements, the bulk samples were used in the same form as for TMA measurements.

## Results

For bulk GeSe_2_ samples, the crystallization process was followed above the glass-forming temperature (*T*_g_) that was found to be 676 K, which is in good agreement with 673 K from the literature.^14,23^ Crystals started to grow from nuclei randomly distributed through the volume and surface of the amorphous phase. Two crystalline forms (LT- and HT-GeSe_2_), which are typical for this composition, were both observed growing on the surface and in the volume of bulk samples, in a temperature interval of 715–830 K. Both crystal types resembled the spherulitic structure with some minor differences. Crystals of HT-GeSe_2_ modification are formed by rectangular needles growing from a single nucleus, as seen in Figure 1a, which is then reformed into a spherulitic crystal shown in Figure 1b. This modification is significantly different in size from LT-GeSe_2_, which is apparent from Figure 1a. The size difference results from the higher growth rate of HT-GeSe_2_ crystals, as is apparent from Figure S1 in the Supporting Information and will be discussed later. LT-modification forms crystals with a spherulitic shape from the beginning of growth, as seen in Figure 1c. The spherulites were built from thin needles, similar to HT-form. It is important to note that both modifications grew on the surface and in the volume of bulk samples. In thin films, only the LT-GeSe_2_ modification was found (Figure 1d–f). The crystal growth rate in TF was significantly higher than in bulk samples (Figure S1 and Table S1 in the Supporting Information), which allowed us to observe the crystal growth far below TF‘s glass transition temperature (*T*_g_ = 663 K).

**Figure 1:**
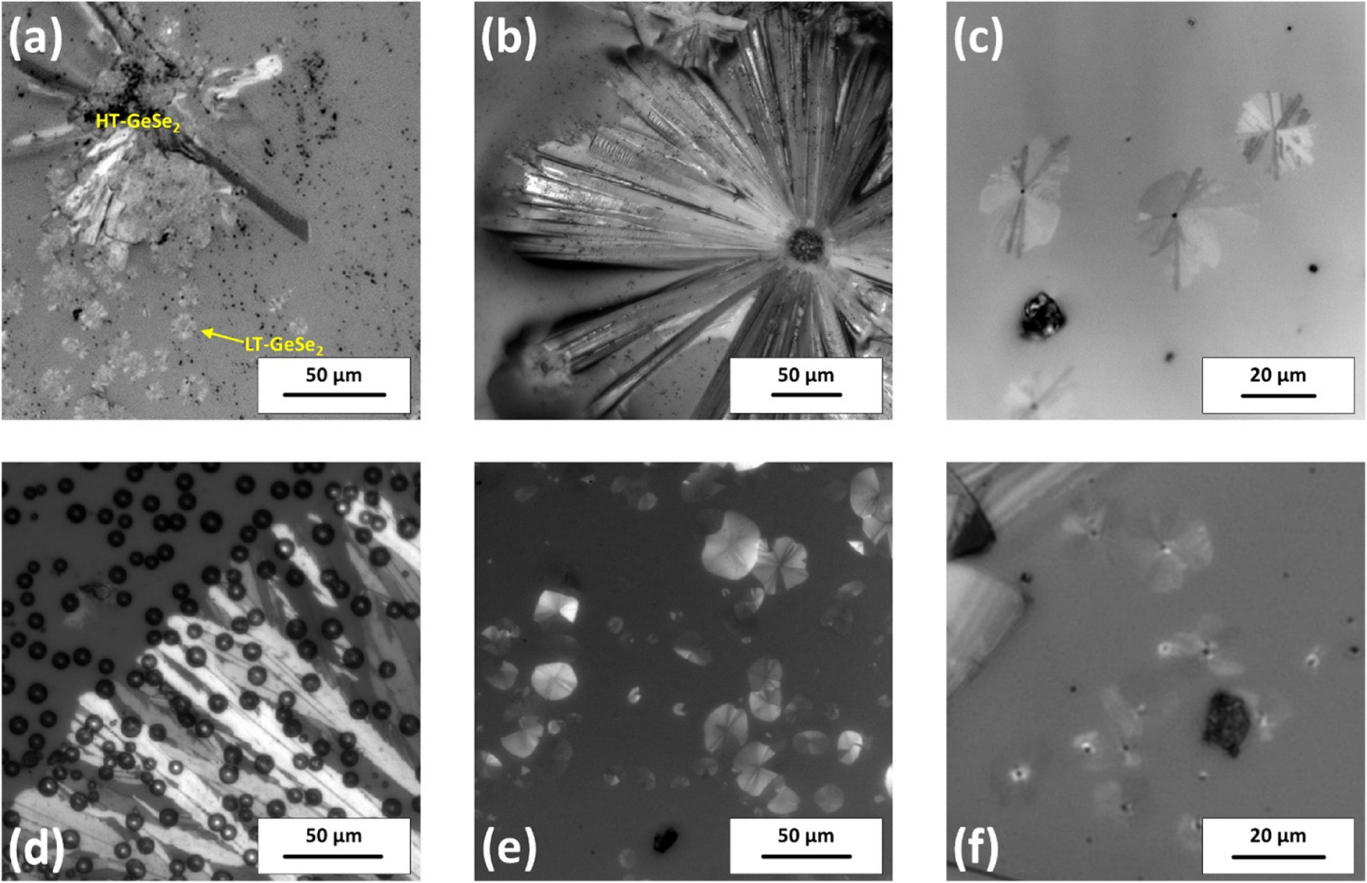
Bulk samples with a growing crystal of (a) both HT- and LT-GeSe_2_ growing at 763 K, (b) HT-GeSe_2_ at 808 K, and (c) a close-up of LT-GeSe_2_ crystals at 728 K. Crystals growing in thin film samples at (d) 703 K (the small circles correspond to the bubbles formed in TF at higher temperatures probably due to detaching of the film from the glass-substrate), (e) 583, and (f) 508 K.

Bulk samples were prepared by cutting as-prepared GeSe_2_ ingots into small pieces with a size of approximately 2 mm. Thin films were cut with a diamond cutter into smaller plates. All samples (thin films and bulks) were then annealed under isothermal conditions in a controlled furnace for chosen time intervals. After that, the samples were rapidly cooled to room temperature. After annealing, the bulk samples were gently cut in half to observe the crystals growing in volume, but the surface crystals were also studied. Then, using the infrared microscope, at least ten isolated crystals or crystal structures were measured as the maximal size of the formed crystals. Thin film samples were measured ex situ and in situ. For the ex situ experiments, one sample was annealed in a furnace for a certain period, after which the sample was removed and cooled to room temperature. Then, the sample was examined under the microscope. The same sample was then put back into the furnace to continue annealing, and the procedure was repeated several times. Such a procedure allowed us to find and observe the evolution of the same crystals during the growth process. Regarding the in situ experiments, the samples were annealed in a temperature-controlled chamber directly coupled with the microscope. Such an arrangement allowed observing a specific area on the sample, and the crystal growth was recorded. The crystals mainly grew from randomly distributed nuclei in the specimen. Nevertheless, crystals were also formed on the edges of some defects (scratches, holes, and broken edges of the sample). Still, only the first case of crystals was suitable for growth measurements due to their spatial separation. The crystal growth behavior shows a linear dependence of crystal length on time, characteristic of interface-controlled kinetics, as shown in Figure S1 in the Supporting Information. Because of that, the crystal growth rate could be easily determined from the slopes of these linear dependencies.

Values of temperature-dependent crystal growth rates in the bulk samples and in thin film samples are presented in Figure 2 and listed in Table S1 in the Supporting Information. It is apparent that the HT-GeSe_2_ crystals, which are present in the bulk volume, grow much faster than the LT-GeSe_2_ crystals. However, with rising temperature, the growth rate difference between the two modifications starts to narrow (*u*_HT_/*u*_LT_ ≅ 40 → 15 for the presented temperature range). For the surface crystals growing in thin film samples, the difference is much larger when compared with HT-GeSe_2_ (*u*_TF_/*u*_HT_ ≅ 440) or even with LT-GeSe_2_ (*u*_TF_/*u*_LT_ ≅ 15800) for a short range where the temperatures overlap.

**Figure 2:**
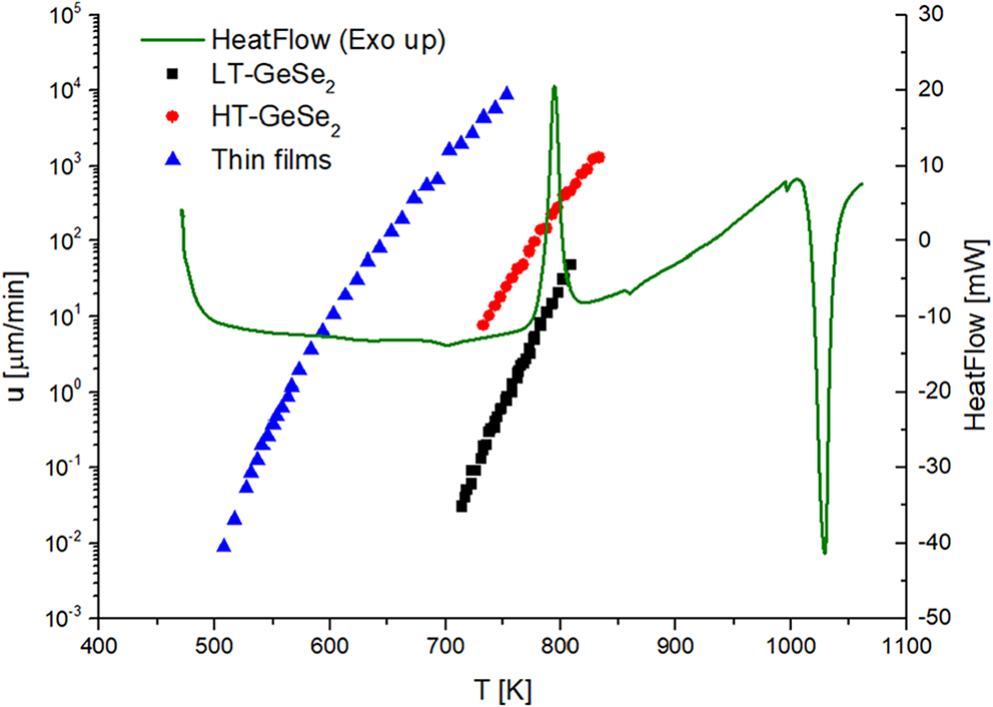
Temperature dependence of the isothermal crystal growth rates of HT-GeSe_2_ (red circles) and LT-GeSe_2_ (black squares) crystals in the bulk glass and LT-GeSe_2_ crystals at the thin film surface (blue triangles). DSC curve of GeSe_2_ bulk glass (green line).

Using DSC, the heat flow that evolved during crystallization can be detected. DSC experiments were performed on samples in the form of bulk, powder, and scratched thin film, and measured in standard aluminum pans. By integrating heat flow over the crystallization peak, we can determine the enthalpy change of the crystallization process, Δ*H*_c_. Δ*H*_c_ was found to be in the range of −13.5 to −14.9 kJ/mol. These values are comparable with an enthalpy of devitrification of −15.0 ± 0.1 kJ/mol, obtained from the literature.^24^ The DSC measurements were also performed in open silica ampules that allowed us to study the bulk samples up to 1073 K. The obtained DSC data on a bulk sample measured in an open silica ampule are plotted in Figure 2, which describes the temperature range from well below the glass transition temperature to temperatures above the melting point of the GeSe_2_ crystal. In such a case, the melting of GeSe_2_ could be followed, and the parameters of the melting were estimated. The enthalpy of melting (Δ*H*_m_) was found to be 23.3 kJ/mol, and the onset of the melting peak (*T*_m_) was found to be 1005 K. These values correspond well with those estimated in the work of Stølen et al. (Δ*H*_m_ = 24 kJ/mol and *T*_m_ = 1009 K).^8^

Viscosity dependence on temperature was studied in the prepared bulk glasses and thin films. The as-prepared GeSe_2_ bulk glass was cut into small pieces approximately 6 mm × 6 mm wide and 2.5 mm thick, which were ground to ensure perfectly flat and parallel surfaces. These samples were measured using the penetration method of TMA, which was used to measure viscosities in the range of 10^8^ – 10^13^ Pa·s. The samples were penetrated with hemispherical and cylindrical indenters. The near-surface viscosities of bulk samples were obtained using the nanoindentation (NI) method using the sapphire Berkovich indenter. All measured experimental viscosity values, with experimental errors less than 0.1 Pa·s, are plotted in Figure 3 and listed in Table S2 in the Supporting Information. Unfortunately, the viscosities in TF samples could not be obtained by NI due to the fast crystallization of the samples, even close to and below the *T*_g_.

**Figure 3:**
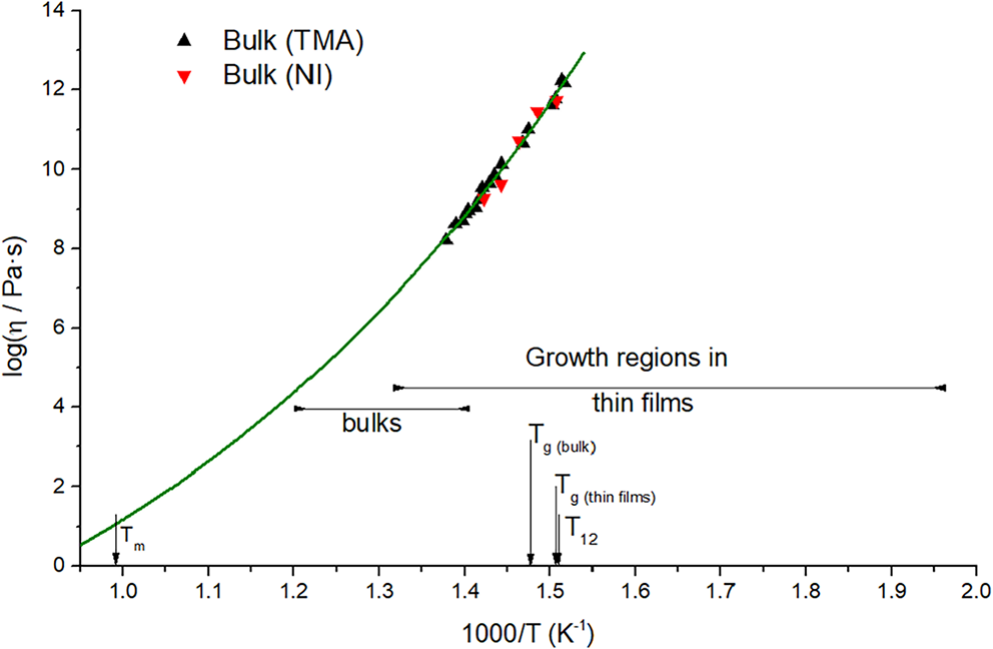
Temperature dependence of the viscosity of bulk samples measured with TMA (black points) and measured with nanoindentation (NI - red points), described by the MYEGA viscosity model (green line).

## Discussion

### Viscosity Behavior

The viscosity values of bulk samples measured by TMA and NI are identical within experimental error. Because of that, these methods are comparable to each other, and the results can be unified. The temperature dependence of viscosity measured in this paper can be described using the Mauro-Yue-Ellison-Gupta-Allan (MYEGA) equation^25^

1 $$\log\left(\eta \right)=\log\left(\eta_{0}\right)+\left[12-\log\left(\eta_{0}\right)\right]\cdot \left(\frac{T_{12}}{T}\right)\cdot \exp[\left(\frac{m}{12-\log\left(\eta_{0}\right)}-1\right)\cdot (\frac{T_{12}}{T}-1)]$$

where η is the measured shear viscosity, η_0_ is the extrapolated viscosity at infinite temperature, *T*_12_ is a parameter that describes at what temperature the shear viscosity gains a value of 10^12^ Pa·s (also known as the viscosity glass-forming temperature), and *m* is the fragility of the material at the temperature *T*_12_. The MYEGA equation was chosen based on our previous results in the Ge–Se system.^26,27^ Because of the lack of experimental data in the melt region, the value of log­(η_0_) was chosen to be −5 according to Angell’s work.^28^ Parameters calculated from eq 1 and the measured viscosity data of GeSe_2_ are log­(η_0_/Pa·s) = −5, *T*_12_ = 661.9 ± 0.8 K, and *m* = 47.2 ± 1.1. The value of the fragility parameter *m* is relatively low, making the germanium diselenide a relatively strong chalcogenide glass-forming liquid, comparable to As_2_Se_3_ or As_2_S_3_.^29,30^

Unfortunately, the viscosity behavior in thin films could not be followed due to fast crystallization. Nevertheless, our previous study on Ge_25_Se_75_ showed that the viscosities in bulks and thin films were identical within the experimental error.^26^ Since the composition of Ge_25_Se_75_ and GeSe_2_ is close, and both materials are built by homogeneously distributed edge- or corner-shared GeSe_4_ tetrahedra (interconnected by Se-chains in Ge_25_Se_75_),^12,13^ we can assume a similar behavior also for GeSe_2_. Therefore, we use the same MYEGA parameters to estimate viscosities in both bulks and thin films in the temperature range where crystal growth occurred.

### Crystal Growth Kinetics

The Arrhenius model is the simplest model that can describe the temperature dependence of the crystal growth rate, providing information about the apparent activation energy of crystal growth, *E*_G_. The crystal growth of GeSe_2_ was measured in a relatively narrow temperature range, where the data plot could be assumed to be of a simple exponential dependence. Therefore, the linearized Arrhenius equation could be used in the form

2 $$\ln\left(u\right)=\frac{1}{T}\cdot \left(-\frac{E_{G}}{R}\right)+\ln\left(A_{f}\right)$$

where *u* represents the isothermal crystal growth rate at the temperature *T*, *R* is the gas constant, and *A*_f_ is the pre-exponential factor. From the slope of the dependence of ln­(*u*) on 1/*T*, which can be seen in Figure 4, the activation energy of crystal growth (*E*_G_) could be estimated. For thin film samples, this value was found to be 174.2 ± 1.3 kJ/mol, whereas the bulk samples have shown much larger values, for HT-GeSe_2_ equal to 267.6 ± 2.6 kJ/mol and LT-GeSe_2_ equal to 363.9 ± 2.9 kJ/mol. These values are also higher than the reported apparent activation energy (204 ± 7 kJ/mol) obtained from DSC data for GeSe_2_ glass powder.^31^ The obtained activation energies provided the expected differences. When the *E*_G_ represents an apparent energy barrier for crystal growth, the smaller the *E*_G_, the faster crystal growth can be expected.

**Figure 4:**
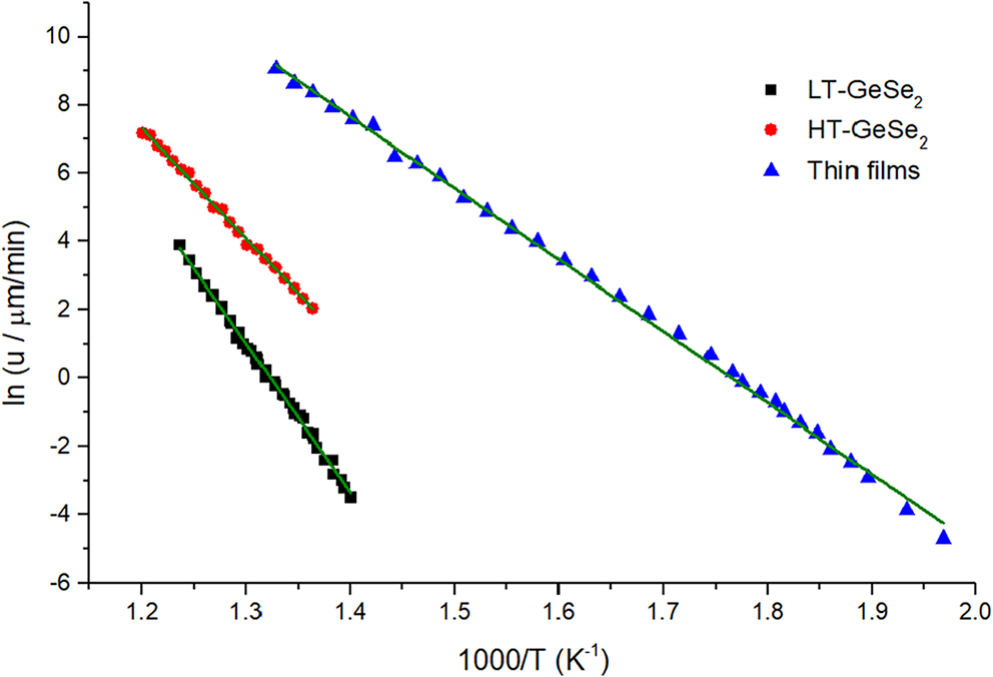
Dependence of ln­(*u*) vs 1000/*T*. From the slopes of these dependencies, the activation energies of crystal growth were calculated for the thin films and bulk samples.

Although the Arrhenius model is the simplest crystal growth model, it is not applicable to describe and predict crystal growth rates in a wide temperature range, especially in the whole region of the undercooled melt, from the glass transition temperature to the melting point. Therefore, an appropriate model must be found. From the linear dependence of crystal length on time, shown in Figure S1 in the Supporting Information, it is apparent that the crystal growth rate is constant at a given temperature. Because of that, the crystal growth is controlled by the kinetics of the liquid-crystal interface. In our previous works,^32–34^ the application of temperature dependence of viscosity (η), change in Gibbs free energy between amorphous and crystalline phases (Δ*G*), and parameter ξ was shown to assess an appropriate crystal growth model. The parameter ξ represents a decoupling between viscosity and diffusion coefficient (*D* ≈ η^–ξ^)^35^ and can be estimated from the kinetic part of the crystal growth rate (*u*_kin_)­

3 $$u_{kin}=\frac{u}{1-\exp\left(-\frac{\Delta G}{R\cdot T}\right)}\propto \eta^{-\xi }$$

When the heat capacities of the crystalline and amorphous phases are not known, the Δ*G* can be expressed by different approximations (e.g., Turnbull,^36^ Hoffmann,^37^ Thompson and Spaepen,^38^ etc.). Malek et al.^39^ showed in their work on crystal growth in GeS_2_ glass that the model proposed by Thompson and Spaepen^38^ provides a reasonable description of the Δ*G* dependence on temperature comparable to that obtained directly from heat capacities. Since the GeS_2_ and GeSe_2_ chalcogenide glass formers are structurally very similar, the same approach is used in this study. The Thompson and Spaepen^38^ approximation is given as

4 $$\Delta G=\frac{\Delta H_{m}\cdot \Delta T}{T_{m}}\cdot \left(\frac{2\cdot T}{T_{m}+T}\right)$$

where Δ*H*_m_ stands for melting enthalpy, *T*_m_ is the melting temperature, and Δ*T* is undercooling, expressed as Δ*T* = *T*_m_ – *T*. On behalf of these parameters, the parameter ξ could be estimated by plotting the dependence of log *u*_kin_ on log η (Figure 5).

**Figure 5:**
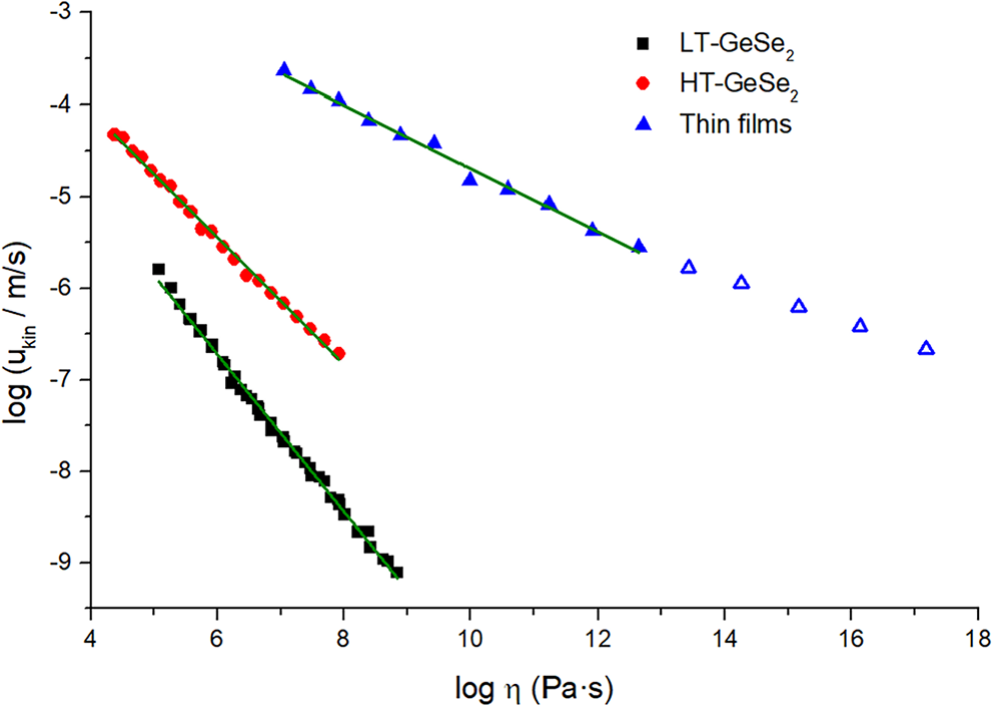
Linearized dependence of log­(*u*_kin_) on log­(η) as a method of estimating the decoupling parameter ξ for HT-GeSe_2_ (red circles), LT-GeSe_2_ (black squares), and thin films (blue triangles; empty triangles represent data below *T*_12_).

Due to high crystal growth rates in TF, the crystal growth in TF could be followed far below the *T*_g_, as is apparent from Figure 3. Using the estimated MYEGA equation for temperature below *T*_g_ would have led to incredibly high, unrealistic values of viscosity (over 10^34^ Pa.s). Therefore, only the data up to the viscosity of 10^18^ Pa.s (about 50 K below *T*_g_) were implemented in Figure 5 to assess the relation between *u*_kin_ and η below *T*_g_.

The log *u*_kin_ vs log η dependence for thin film samples is not linear in the whole measured range, but it is rather divided into two linear dependencies, with a breakpoint at the viscosity of 10^12.5^ Pa·s. Parameter ξ is therefore equal to 0.34 ± 0.01 for thin films above the *T*_12_. For HT-GeSe_2_ it is then 0.69 ± 0.01 and 0.86 ± 0.01 for LT-GeSe_2_.

Three phenomenological models are usually used to describe the interface kinetics.^40^ These are the Normal growth model, Screw dislocation growth model, and the two-dimensional (2D) surface nucleated growth model. The most appropriate model for a given set of experimental kinetic data can be chosen based on the dependence of reduced crystal growth rate *U*_R_ on undercooling Δ*T*.^41,42^ Reduced crystal growth rate can be expressed with respect to the decoupling parameter ξ in the following form

5 $$U_{R}=\frac{u\cdot \eta^{\xi }}{1-\exp\left(-\frac{\Delta G}{R\cdot T}\right)}$$

For the calculation of *U*_R_, the crystal growth rates *u* with corresponding temperatures *T* are needed (Table S1 in the Supporting Information). The temperature dependence of viscosity η can be expressed by the MYEGA equation using parameters listed below eq 1 in the previous section of this paper.

Three different plots of *U*_R_ on Δ*T* for thin film samples and both HT and LT modifications growing in bulk samples are presented in Figure 6. The constant character of all three data sets suggests the normal growth model.^41,42^ The normal growth model is expressed by the following equation^40^

6 $$u_{r}\left(T\right)=\frac{u\left(T\right)}{2}=\frac{k_{B}\cdot T}{3\cdot \pi \cdot a_{0}^{2}\cdot \eta^{\xi }}\cdot [1-\exp\left(-\frac{\Delta G}{R\cdot T}\right)]$$

where *u*_r_(*T*) is the crystal growth rate measured in one direction (as an increase of the spherulitic crystal’s radius). Since the crystal growth rate *u*(*T*) in this work was measured as a change of maximal length of the formed crystals (in two directions as a mean diameter), the obtained growth rate values need to be divided by 2. *k*_B_ is the Boltzmann constant, and *a*_0_ is the mean distance between atoms in the interface layer.

**Figure 6:**
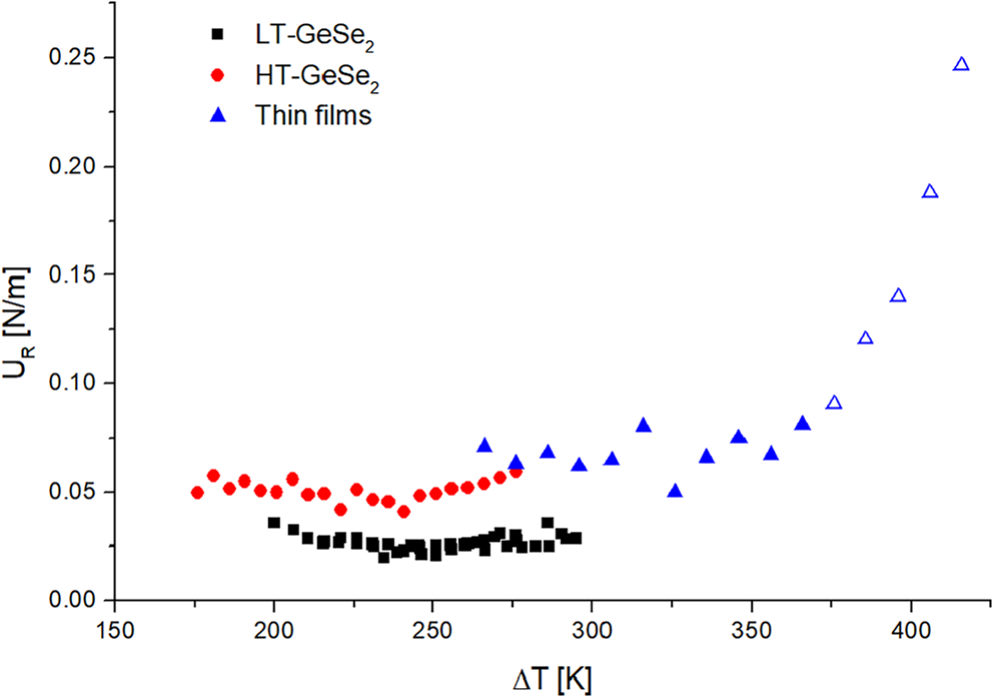
Reduced crystal growth rate dependence on undercooling for HT-GeSe_2_ (red circles), LT-GeSe_2_ (black squares), and thin film samples (blue triangles, empty points correspond to the data below *T*_12_).

With all of the necessary parameters known, the kinetic data of crystal growth in bulk and thin film samples were described with a normal growth model (eq 6), which is shown in Figure 7. The thin film samples were described only in the area above the *T*_12_, as was also done for the evaluation of parameter ξ described earlier. The reason is that the growth rate values in the measured temperature range below *T*_12_ would correspond to viscosity values from 10^12^ to 10^34^ Pa·s. Since the viscosities over 10^13^ Pa·s are experimentally hardly accessible, the extrapolation of MYEGA (eq 1) would be highly inaccurate. This is obvious from the progress of the kinetic model from *T*_g_ to lower temperatures, where the model differs greatly from the experimental data. Similar results were described for the surface crystallization of indomethacin^43^ or nifedipine^44^ below *T*_g_, where the crystal growth rate had suddenly risen to higher values than expected, which could be connected to the transition from diffusion-controlled to diffusionless crystal growth.^43–46^ In the case of GeSe_2_ thin films, the rise is much smaller, but it is still enough to be observable in the experimental data in Figure 7.

**Figure 7:**
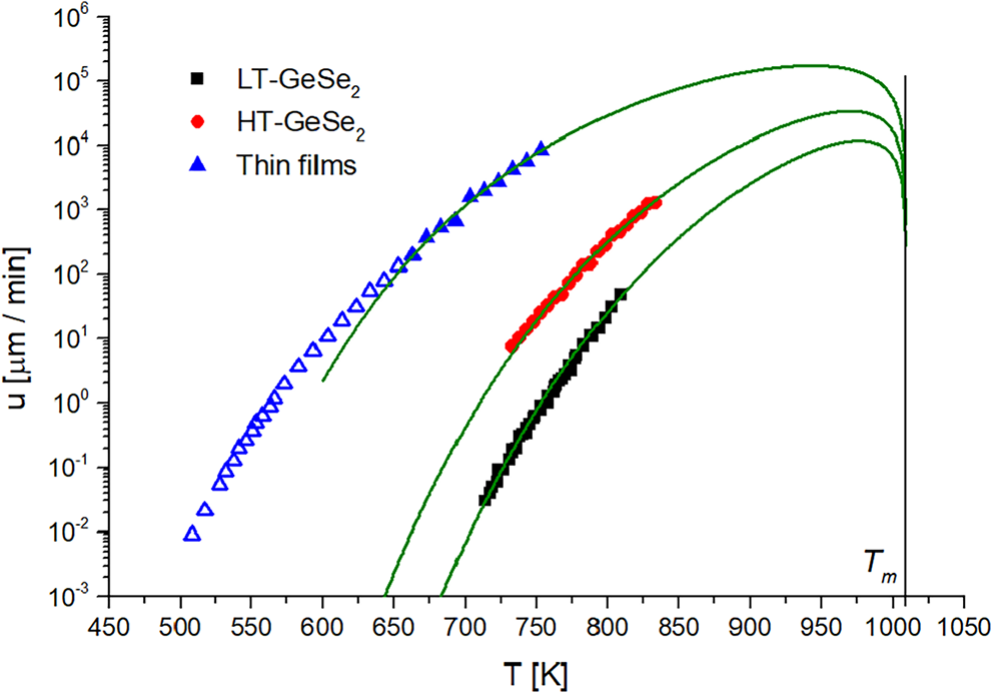
Crystal growth rate dependence on temperature for all measured samples fitted with normal growth kinetic models (lines).

From the fitted models, the parameters *a*_0_ were found to be 1.83 ± 0.11, 2.04 ± 0.11, and 2.56 ± 0.18 Å for thin film samples, HT-GeSe_2_ and LT-GeSe_2_ crystals, respectively. The size of the found parameters *a*_0_ corresponds to the interatomic distance of Ge–Se atoms (2.36 Å) in GeSe_4_ tetrahedra units.^15,47,48^ This correlation might indicate that single GeSe_4_ tetrahedral units are incorporated into the crystal surface during the growth.

### Self-Diffusion in Crystal Growth

The measurement of diffusion coefficients in glass-forming materials is very rare, especially for chalcogenide glasses. The kinetic part of the crystal growth rate, which corresponds to the diffusion coefficient, strongly decouples from the viscosity, especially for thin films. This means that the crystal growth is mainly driven by diffusion instead of viscosity. One of the most common equations in crystal growth theory, used to describe diffusion, is the Stokes–Einstein-Eyring (SEE) equation^49^

7 $$D_{\mathrm{SEE}}\left(T\right)=\frac{k_{B}\cdot T}{a_{0}\cdot \eta }$$

For our experiments, the SEE equation cannot describe the data well, which was also shown in the literature for chalcogenide,^32,34,50,51^ oxide,^52^ and molecular^35,53^ glasses, where the SEE usually cannot describe the diffusion process at lower temperatures, especially for surface crystallization, or thin film crystal growth. As mentioned earlier, growing GeSe_2_ crystals are formed from GeSe_4_ tetrahedral units. These tetrahedra are then transported through the amorphous medium to the interface of growing crystals. The transportation process could therefore be described by an effective diffusion coefficient, which is estimated based on the size of the structural unit. The effective diffusion coefficient can be calculated directly from the experimental data, making it independent of the previous relation (eq 7)^40–42^

8 $$u_{r}=f\cdot \frac{D_{eff}}{a_{0}}\cdot \left[1-\exp\left(-\frac{\Delta G}{R\cdot T}\right)\right]$$

where *f* defines the growth mechanism, which is equal to *f* = 1 for the chosen normal growth model within this work. With the use of parameter *a*_0_, calculated from the normal growth model, the coefficient of effective self-diffusion *D*_eff_ can be estimated. Figure 8 shows the estimated data of *D*_eff_. The transport of the structural units in the amorphous material was fastest in thin film samples, which was expected from the high crystal growth rates. The high speed of the structural units’ transport in TFs may originate from internal stresses in the TF or between TF and the substrate, as well as from the different structure (in comparison to bulk samples), or from a migration of foreign ions diffusing from the substrate.^54^ Similar behavior was also reported for crystal growth in GeS_2_.^39,51^

**Figure 8:**
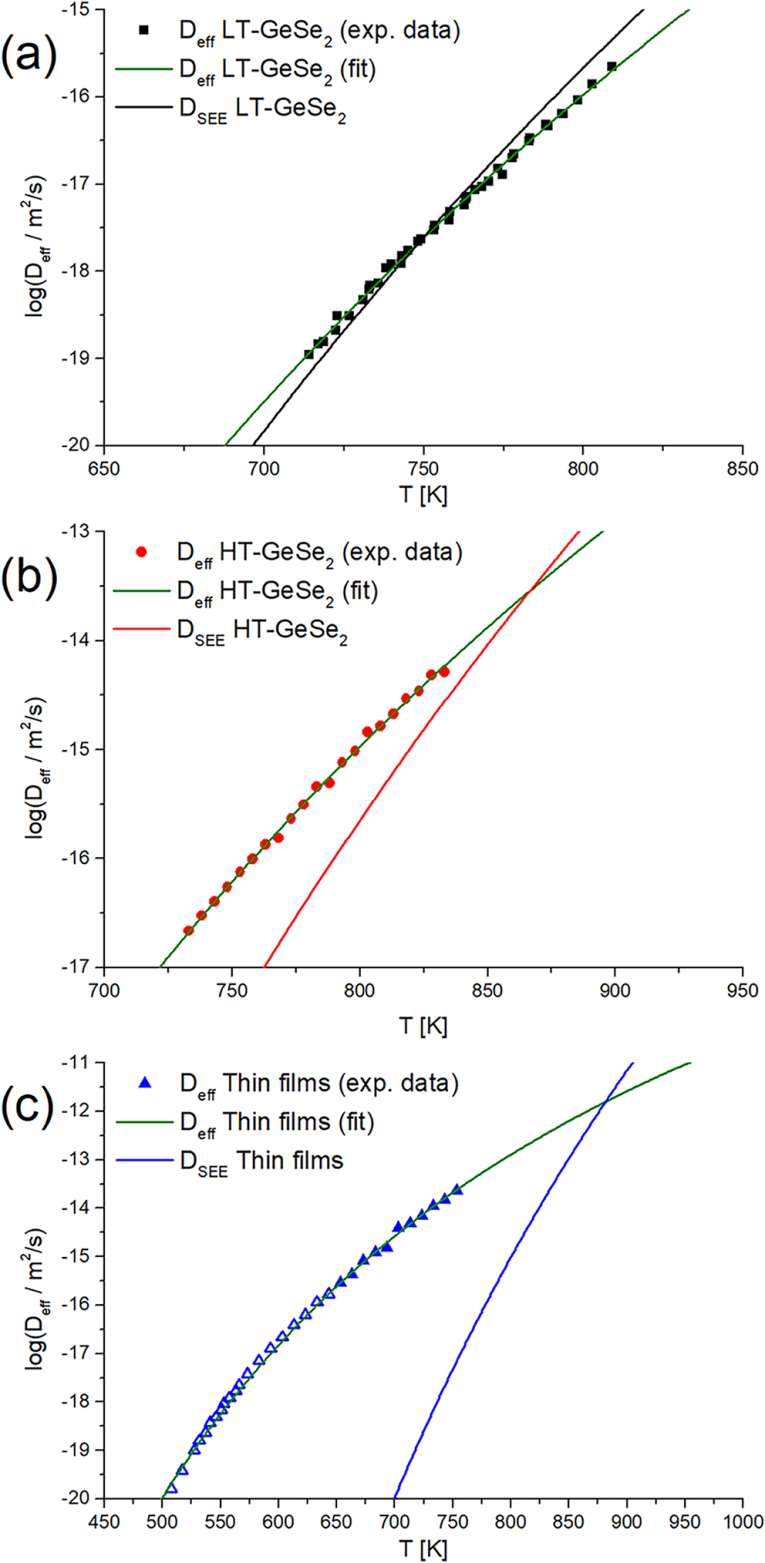
Temperature dependence of diffusion coefficient in GeSe_2_ bulk samples for (a) LT, (b) HT modification, and (c) thin films. Green lines show the Arrhenian fit of *D*_eff_. Black, red, and blue lines represent the Arrhenian fits of *D*_SEE_.

On the other hand, the explanation of differences in *D*_eff_ in bulk samples is challenging. Both the crystal forms (HT- and LT-GeSe_2_), as well as the amorphous phase, are formed from the same GeSe_4_ tetrahedra units. Therefore, one could expect that the transport rate of the structural units would be similar. Nevertheless, the data shown in Figure 8 provide *D*_eff_ values about 1–1.5 orders of magnitude higher for the HT-GeSe_2_ than for the LT-GeSe_2_. The reason for such a difference is unknown. The difference in the *D*_eff_ values for HT- and LT-GeSe_2_ form might be connected to the structure of the growing crystals.^9,10,15,17,18^ LT-GeSe_2_ crystals are formed from only corner-shared GeSe_4_ tetrahedral units and are considered to have similar structures to LT-GeS_2_ crystals.^10,11^ These crystals consist of two kinds of linear chains that are not included in one plane. Therefore, they form a three-dimensional structure.^10,11,17,18^ On the other hand, both the HT-GeSe_2_ and HT-GeS_2_ crystals form a layered two-dimensional structure. These crystals consist of corner-sharing tetrahedral chains connected like a ladder by bridges made of a pair of edge-sharing tetrahedra.^10,15^ The differences in the crystal structure of LT- and HT-GeSe_2_ might have a significant impact on the attachment of the GeSe_4_ tetrahedral unit to the growing crystal, resulting in differences in crystal growth rates or effective diffusion coefficient.

The temperature dependence of *D*_eff_ is compared to the diffusion coefficient calculated from the SEE eq (eq 7), expressed here as *D*_SEE_. The data of *D*_eff_ are described by the Arrhenius-type plot to show a possible extrapolated trend for a broader temperature range. This description is useful for the *D*_eff_ vs *D*_SEE_ comparison, visible in Figure 8, since it shows us the scale of the decoupling between *D*_eff_ and *D*_*S*EE_ for all three plotted kinetic data. It is also obvious that the scale of decoupling is related to the value of the decoupling parameter ξ calculated earlier. For LT-GeSe_2_, *D*_SEE_ is very close to the estimated *D*_eff_ (Figure 8a), since the parameter ξ is close to 1. On the other hand, the decoupling is strong for HT-GeSe_2_ (Figure 8b) and thin film samples (Figure 8c), resulting in the parameter ξ being significantly lower than 1.

The calculated effective diffusion coefficients and their corresponding experimental data of crystal growth rates are shown in Figure 9. This relation allows for comparison of the mentioned D_eff_ with surface self-diffusion coefficients determined for selenium and molecular glass formers,^55^ where they can be compared with the surface self-diffusion coefficients for amorphous selenium and molecular glass formers.^55^ The molecular systems show a great correlation between surface crystal growth rate (*u*_s_) and the surface self-diffusion coefficient, which is expressed as

9 $$u_{s}\approx D_{s}^{0.87}$$

The trend of the effective diffusion coefficient of GeSe_2_ in Figure 9 shows a relation similar to that in eq 9, showing that this equation can also be applied for volume crystal growth in bulk samples and for crystal growth in thin films, since the exponent values are 0.963 ± 0.002, 0.939 ± 0.002, and 0.949 ± 0.002 for LT-GeSe_2_, HT-GeSe_2_, and thin films, respectively. This can also be compared to recently published data of Ge_25_Se_75_ ^26^ with values 0.912 ± 0.004 and 0.863 ± 0.007 for thin films and bulk samples, respectively. The crystal growth rate measurements have, therefore, great potential for providing missing information about the self-diffusion mechanisms in chalcogenide glass.

**Figure 9:**
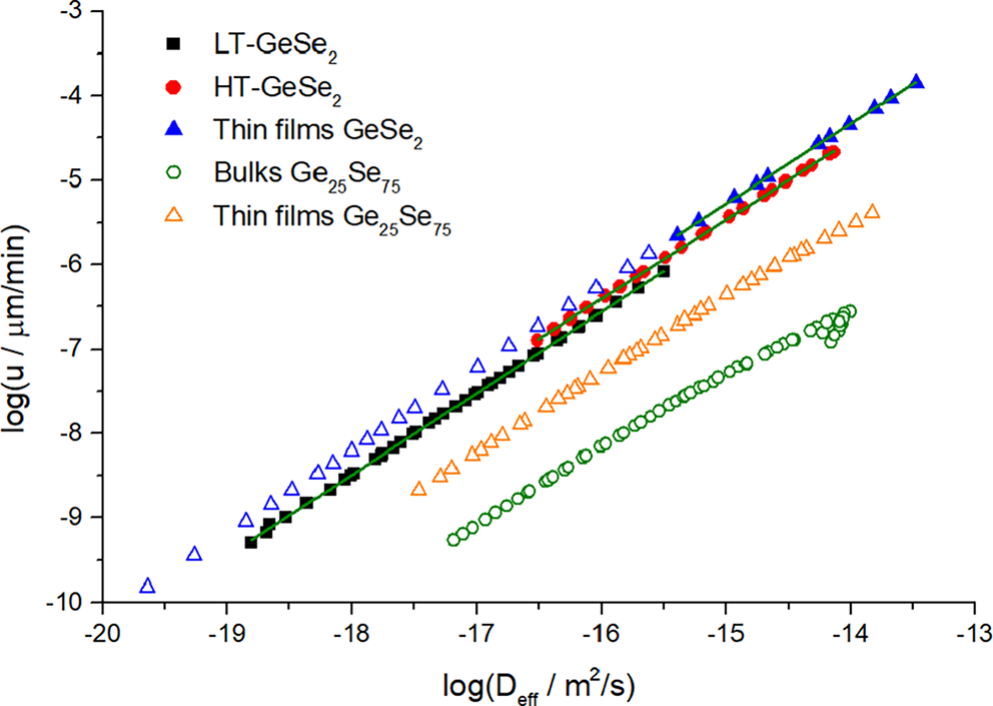
Relation between the effective diffusion coefficient and corresponding crystal growth rates for GeSe_2_, and comparison with recently published data of Ge_25_Se_75_.^26^

## Conclusions

Crystal growth and viscous behavior were studied within this work in an amorphous GeSe_2_ glass former in the form of bulk and thin film (TF) samples. Crystals of low-temperature (LT) GeSe_2_ form growing in thin films showed faster growth rates, allowing for the following crystal growth far below the glass-transition temperature. In bulk samples, two forms of GeSe_2_ crystals were found: low temperature (LT) and high temperature (HT) forms. In all cases, the formed crystals grew linearly with time, indicating a crystal growth driven by liquid-crystal interface kinetics. Systematic analysis of crystal growth and viscosity revealed a significant decoupling from the Stokes–Einstein–Eyring relation between the kinetic part of the crystal growth rate and viscosity. Therefore, corrections for the standard normal growth model were used to describe and extrapolate the crystal growth data in a wide temperature range. The crystal growth model also provided information about the size of the structural units incorporated on the crystal surface during its growth. The size of the structural units corresponds to the interatomic distance of Ge–Se atoms in the GeSe_4_ tetrahedral units.

Effective diffusion coefficients (*D*_eff_) were calculated based on the growth data and the estimated structural units incorporated into the crystal. The crystal growth in thin films provided a higher *D*_eff_ than in bulk samples. The high structural unit transport in TF may originate from internal stresses in the TF or between TF and the substrate, as well as from the different structures (in comparison to bulk samples) or from a migration of foreign ions diffusing from the substrate. Also, the *D*_eff_ values in bulk samples were found to be different for the growth of LT- and HT-GeSe_2_ forms. The differences might be attributed to the different structures of the crystals formed as two-dimensional edge- and corner-shared GeSe_4_ tetrahedra (HT-form) or three-dimensional corner-shared GeSe_4_ tetrahedra (LT-form).

## Supplementary Material



## Data Availability

The data that support the findings of this study are openly available in ZENODO at https://zenodo.org/, reference number 10.5281/zenodo.15737124.

## Notes

Additional experimental details and tables with data on measured viscosities and crystal growth rates (PDF)
