# Supplementary material for: Crystal Growth Kinetics of GeSe2 Polymorphs in Bulk Glasses and Thin Films: Role of Self-Diffusion and Viscosity
Source: Cryst Growth Des. 2025 Sep 13;25(19):8232–40. doi: 10.1021/acs.cgd.5c01063 (PMC12492402; doi:10.1021/acs.cgd.5c01063)
Supplement: Supplementary file 1 [file cg5c01063_si_001.pdf]

Crystal Growth Kinetics of $GeSe_2$ Polymorphs in Bulk Glasses and Thin Films: Role of Self-Diffusion and Viscosity

David Vaculík*, Jaroslav Barták, Simona Martinková, Petr Koštál, Jiri Málek

AUTHOR INFORMATION

Corresponding Author:

David Vaculík - Department of Physical Chemistry, University of Pardubice, Studentska 573, 53210 Pardubice, Czech Republic; ORCID: 0009-0000-9441-907X;
Email: david.vaculik@student.upce.cz

Authors

Jaroslav Barták - Department of Physical Chemistry, University of Pardubice, Studentska 573, 53210 Pardubice, Czech Republic; ORCID: 0000-0001-8675-1144;

Simona Martinková - Department of Physical Chemistry, University of Pardubice, Studentska 573, 53210 Pardubice, Czech Republic; ORCID: 0000-0001-9117-7773

Petr Koštál - Department of Inorganic Technology, University of Pardubice, Doubravice 41, 53210 Pardubice, Czech Republic; ORCID: 0000-0003-1834-9721

Jiri Málek - Department of Physical Chemistry, University of Pardubice, Studentska 573, 53210 Pardubice, Czech Republic; ORCID: 0000-0002-7502-5320

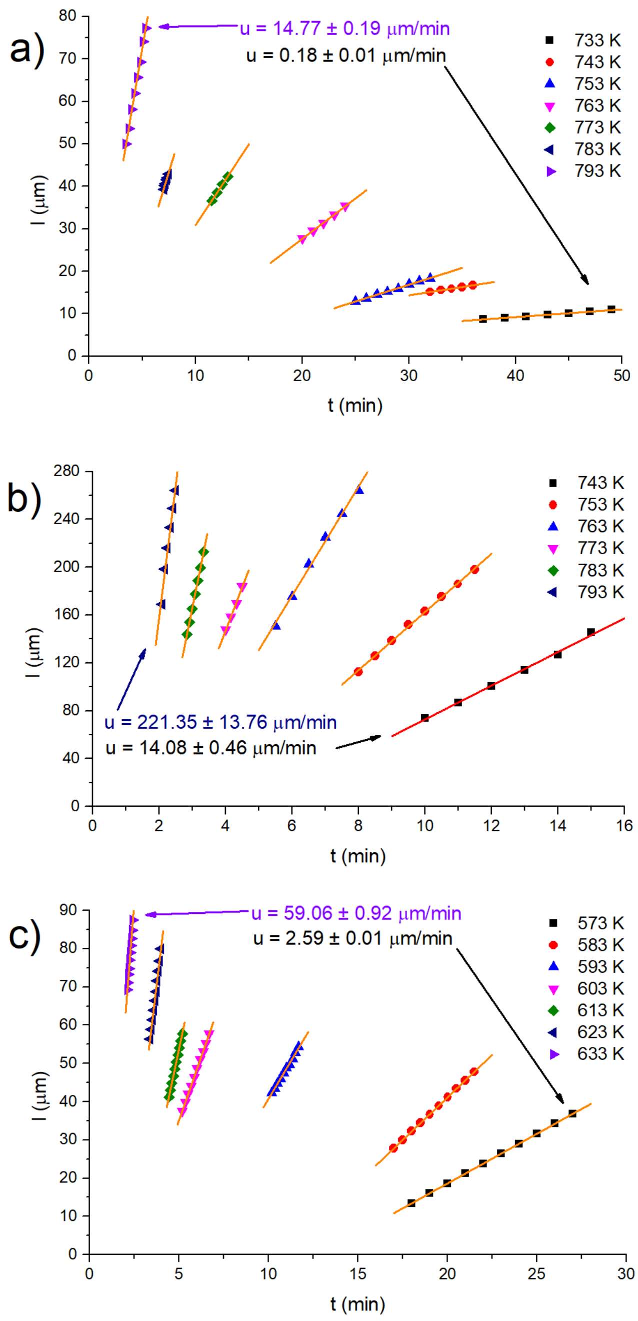

Figure S1 – A selection of linear dependences of the crystal length *l* on time *t* for various temperatures in (a) LT and (b) HT-$GeSe_2$ modification in bulk samples and in (c) thin film samples, with examples of crystal growth rate values.

Table S1 – Crystal growth rate data

Thin films		$HT\text{-}GeSe_2$		$LT\text{-}GeSe_2$	
T [K]	u [μm/min]	T [K]	u [μm/min]	T [K]	u [μm/min]
507.9	0.0090 ± 0.0010	733.2	7.63 ± 0.30	714.1	0.0325 ± 0.0011
517.2	0.0211 ± 0.0022	738.2	10.32 ± 0.59	717.1	0.0425 ± 0.0029
527.6	0.0542 ± 0.0083	743.2	13.8 ± 1.2	718.6	0.04509 ± 0.00053
531.9	0.0861 ± 0.0011	748.2	18.36 ± 0.38	722.6	0.0602 ± 0.0035
537.4	0.1259 ± 0.0035	753.2	25.1 ± 1.4	723.1	0.0891 ± 0.0045
541.3	0.2011 ± 0.0043	758.2	32.5 ± 2.3	726.9	0.0875 ± 0.0019
546.2	0.2641 ± 0.0034	763.2	43.1 ± 1.9	731.2	0.1331 ± 0.0010
550.8	0.3708 ± 0.0014	768.2	48.7 ± 2.3	732.9	0.172 ± 0.048
553.2	0.494 ± 0.035	773.2	72.1 ± 4.2	733.2	0.1930 ± 0.0045
557.9	0.641 ± 0.015	778.2	96.0 ± 6.5	735.9	0.202 ± 0.011
563.2	0.889 ± 0.046	783.2	137.9 ± 8.5	738.2	0.301 ± 0.011
566.2	1.179 ± 0.052	788.2	146.0 ± 8.0	739.9	0.335 ± 0.017
573.2	1.95 ± 0.22	793.2	222 ± 31	742.9	0.338 ± 0.004
583.2	3.67 ± 0.27	798.2	278 ± 26	743.2	0.412 ± 0.042
593.2	6.45 ± 0.95	803.2	405 ± 49	745.2	0.473 ± 0.011
603.2	10.93 ± 0.56	808.2	453.9 ± 4.1	748.2	0.590 ± 0.023
613.2	19.2 ± 1.6	813.2	573 ± 16	749.1	0.622 ± 0.018
623.2	31.0 ± 1.4	818.2	774 ± 21	753.2	0.787 ± 0.016
633.2	54.5 ± 5.8	823.2	893 ± 21	753.5	0.882 ± 0.023
643.2	80 ± 11	828.2	1222 ± 116	758.1	1.008 ± 0.039
653.2	133 ± 17	833.2	1278 ± 47	758.2	1.25 ± 0.15
663.2	196 ± 14			762.7	1.479 ± 0.035
673.2	373 ± 23			763.2	1.73 ± 0.12
683.2	539 ± 41			763.6	1.848 ± 0.012
693.2	653 ± 61			766	2.205 ± 0.033
703.2	1621 ± 28			768.2	2.36 ± 0.18
713.2	1966 ± 99			770.5	2.725 ± 0.046
723.2	2759 ± 105			773.2	3.78 ± 0.19
733.2	4326 ± 137			774.6	3.197 ± 0.076
743.2	5608 ± 73			777.7	4.964 ± 0.050
753.2	8642 ± 299			778.2	5.38 ± 0.15
				783.1	7.56 ± 0.10
				783.2	8.20 ± 0.35
				788.2	11.38 ± 0.81
				788.9	11.07 ± 0.25
				793.2	14.8 ± 1.1
				793.7	14.83 ± 0.65
				798.4	21.1 ± 1.8
				802.8	31.39 ± 0.83
				808.9	48.9 ± 3.2

Table S2 - Experimental values of viscosity

T (K)	Bulk (TMA) log (n / Pa·s)	T (K)	Bulk (NI) log (n / Pa·s)
725.6	8.21	703.2	9.26
720.0	8.62	693.1	9.63
715.7	8.7	683.1	10.73
713.9	8.88	673.2	11.48
712.3	8.96	663.2	11.74
708.1	9.04		
706.1	9.25		
704.5	9.36		
704.2	9.54		
700.0	9.65		
696.9	9.85		
692.8	10.14		
681.0	10.68		
678.1	11.02		
665.4	11.65		
663.7	11.78		
659.1	12.17		
660.6	12.23		
